# Examining US Public Early Intervention for Toddlers With Autism: Characterizing Services and Readiness for Evidence-Based Practice Implementation

**DOI:** 10.3389/fpsyt.2021.786138

**Published:** 2021-12-16

**Authors:** Aritz Aranbarri, Aubyn C. Stahmer, Meagan R. Talbott, Marykate E. Miller, Amy Drahota, Melanie Pellecchia, Angela B. Barber, Elizabeth McMahon Griffith, Elizabeth H. Morgan, Sally J. Rogers

**Affiliations:** ^1^Collaborative START Lab, The MIND Institute, Psychiatry and Behavioral Sciences, University of California, Davis, Sacramento, CA, United States; ^2^Child and Adolescent Mental Health Area, Psychiatry and Psychology, Hospital Sant Joan de Déu Barcelona, Esplugues de Llobregat, Spain; ^3^Child and Adolescent Mental Health Research Group, Psychiatry and Psychology, Institut de Recerca Sant Joan de Déu, Esplugues de Llobregat, Spain; ^4^Department of Psychology, Michigan State University, East Lansing, MI, United States; ^5^Center for Mental Health, Psychiatry Department, University of Pennsylvania, Perelman School of Medicine, Philadelphia, PA, United States; ^6^Department of Communicative Disorders, University of Alabama, Tuscaloosa, AL, United States; ^7^Department of Pediatrics, University of Colorado School of Medicine, Aurora, CO, United States; ^8^College of Education, California State University, Sacramento, CA, United States

**Keywords:** ASD, autism, early intervention, community-based research, implementation science, health services

## Abstract

As the rates of Autism Spectrum Disorder (ASD) increase and early screening efforts intensify, more toddlers with high likelihood of ASD are entering the United States' (US') publicly funded early intervention system. Early intervention service delivery for toddlers with ASD varies greatly based on state resources and regulations. Research recommends beginning ASD-specific evidence-based practices (EBP), especially caregiver-implemented intervention, as early as possible to facilitate the development of social-communication skills and general learning. Translating EBP into practice has been challenging, especially in low-resourced areas. The main goal of this study was to obtain a more comprehensive understanding of public early intervention system structure, service delivery practices, and factors influencing EBP use for children with ASD in the US. Participants (*N* = 133) included 8 early intervention state coordinators in 7 states, 29 agency administrators in those states, 57 early intervention providers from those agencies, and 39 caregivers of children with ASD receiving services from those providers. Online surveys gathered stakeholder and caregiver perspectives on early intervention services as well as organizational factors related to EBP implementation climate and culture. Stakeholders identified key intervention needs for young children with ASD. In general, both agency administrators and direct providers reported feeling *somewhat effective* or *very effective* in addressing most needs of children with ASD. They reported the most difficulty addressing eating, sleeping, family stress, and stereotyped behaviors. Data indicate that children from families with higher income received significantly higher service intensity. While administrators and providers reported high rates of high-quality caregiver coaching (>60%), caregivers reported low rates (23%). Direct providers with more favorable attitudes toward EBP had greater EBP use. In turn, provider attitudes toward EBP were significantly associated with implementation leadership and culture at their agency. Results suggest that publicly funded early intervention programs in the US require additional resources and training for providers and leaders to support improved implementation climate and attitudes toward ASD EBPs. Results also suggest that more state system support is needed to increase use of ASD-specific EBP use, including high-quality caregiver coaching, to better serve toddlers with ASD. Recommendations for implementation strategies are addressed.

## Introduction

Autism Spectrum Disorder (ASD) is one of the most common forms of neurodevelopmental disabilities, with a rate of 1 in every 54 children born in United States (US) (1). Increases in awareness and screening have led to a higher demand for autism-specific early intervention services. This has led to a need to better understand how public service systems address early intervention for toddlers with ASD who have delays across multiple areas of development (2).

Research demonstrates that specific early intervention models can lead to significant gains in social communication, language development, and adaptative behavior in young children with ASD (3–6). Several groups have published recommendations and quality indicators for best practices in early intervention for ASD (7) that include using evidence-based approaches, beginning intervention as early as possible, active involvement of caregivers as part of the intervention, individualizing treatment based on child, family, cultural, and contextual needs, using curriculum content with a focus on child's social communication, play skills, cognitive, self-help, and behavioral needs, and providing high levels of staff education and training. Recent studies strongly support the role of caregivers' active involvement in early intervention for achieving optimal short and long-term outcomes (8). Toddlers with ASD may also require a higher intensity of service provision to optimize outcomes (9), although the specific number of hours per week needed is not clear (10, 11).

Despite broad agreement on most of these recommendations, in practice meeting these standards within the available publicly funded early intervention community service system remains very challenging. A recent meta-analysis (12) found less favorable outcomes when children with ASD received community intervention compared to hospital/University-based intervention demonstrating significant differences between the types of services being tested and recommended by researchers, and the community services most families receive. Challenges in community implementation may be related to many variables: the complexity of ASD-specific evidence-based practices (EBP), limited opportunities for and variability in staff training, lack of autism-specific support, large caseloads and high overall work demands, low-intensity of service delivery, high diversity both clinically and culturally among clients and areas served, and low funding rates, among others. However, we have limited information about the specific barriers that limited implementation of EBPs in community early intervention services. To bridge the gap between research and practice, researchers must first understand the implementation context.

In the US, children under the age of 3 with an ASD diagnosis or early signs of ASD are typically eligible for public early intervention services provided by Part C of the Individuals with Disabilities Education Act (13). Few states have clear policies or practices in place regarding the type or intensity of Part C early intervention services for young children with ASD, and only a quarter of states have specific intervention guidelines (14). Services may range from simple surveillance, such as a monthly visit from a social worker to intensive interventions, such as 20 h a week of intervention involving delivery of EBPs and parent education. The average service intensity in Part C is 90 min per week (15). As a result of these variables high-quality ASD-specific practices are especially difficult to access in low-resource areas of the US (16). For example, although Part C requirements prioritize and mandate family involvement in early intervention, existing data indicate that most community providers have caregivers playing a passive rather than an active, collaborative and participatory role in their child's intervention (17–21). This lack of active capacity building for primary caregivers allows for little carryover of intervention strategies into daily routines and does not accomplish the Part C goal of building early intervention competence in the child's family (22).

To better understand how to improve translation of EBP, such as parent coaching, into publicly funded early intervention services, we must identify the current service landscape at multiple levels and from varied perspectives (23). Factors related to organizational leader, direct service provider, and consumer characteristics, as well as the organizational climate for innovation, are all related to the quality and use of EBP. The recent field of implementation science provides guidance for identifying determinants of high-quality use of EBP to guide training, adaptation, and implementation of innovative EBP.

For example, direct service providers report that intervention practices developed in research settings are too rigid and do not serve the diversity and complexity of day-to-day practices (24, 25). This is concerning as data indicate that providers' perceptions toward EBPs are linked to uptake and delivery (26). Thus, practitioner attitudes toward EBPs have been considered a target mechanism to improve EBP implementation (27, 28). Data from one early study indicated early intervention providers working with children with ASD had more favorable attitudes toward EBPs than mental health professionals generally and perceived less divergence between their current practice and EBP (29). However, to the best of our knowledge no recent studies have specifically examined provider attitudes toward the use evidence-based early intervention strategies for ASD, including caregiver involvement in intervention, or whether direct providers are considering the evidence-base of their practices when intervening with their clients (14, 30).

So far, a majority of implementation work has focused primarily on direct service providers as the end-users of EBPs, and less on other individuals involved in community implementation (31). However, implementation science has identified leadership as a key component of successful EBP adoption, implementation, and sustainment in community services (32). Leadership can drive EBP implementation through fostering an organizational context in favor of EBP use, for example, by prioritizing provider access to EBP training. Leaders can be instrumental in institutionalizing EBPs, allocating resources strategically to ensure continuity of implementation, or by serving as EBP champions (32). Therefore, leaders can have a profound influence on both the organizational climate (i.e., staff perception of their work environment) and culture (i.e., normative beliefs and shared behavioral expectations in an organizational unit), which in turn can shape the perceptions, attitudes, and implementation by direct service providers (33).

Overall, there are limited data regarding implementation of EBP in community-based early intervention settings, particularly for families in low-resource areas and from historically marginalized backgrounds. Preliminary data indicate that providers report implementing broader elements of EBP strategies rather than the specific techniques that underlie each EBP, adapting them in various ways to meet child and family needs as they deem appropriate (34). Thus, there is a need to describe the early intervention services taking place for toddlers with an elevated likelihood of ASD, and to examine the organizational context that could support use of EBP in low-resourced community settings.

The current study adds to the small body of the literature in this area by studying the structure and practices involved in community Part C delivery in the US public early intervention system focusing on services for children with or at high likelihood of having ASD living in low-resourced areas. Specifically, we aimed to: (1) characterize early intervention services for ASD across seven states serving families in low-resource areas in the US; (2) examine intervention practices and strategies and use of EBP in these systems; and (3) examine organizational and contextual factors influencing system readiness for EBP implementation.

## Materials and Methods

The survey described in this study was conducted as part of a larger community-partnered project designed to adapt an evidence-based early intervention for use in low-resourced service systems. The study used a community-based participatory research methodology (35) with partners in seven states. Partner groups included a mix of representatives including researchers, early intervention agency administrators and direct service providers, and caregivers of children with ASD participating in the early intervention system in their state. The teams met to identify methods for supporting services in rural and low-resource communities within their state and to collaborate on survey development, recruitment, and data interpretation. This specific study involved surveying early intervention stakeholders at multiple levels of the Part C delivery structure.

### Recruitment and Distribution

Participants included individuals involved in one of four distinct tiers of the federally funded (Part C of the IDEA) early intervention delivery structure in the US, specifically providing services for children with or at high likelihood of having ASD. Inclusion criteria were as follows: (a) State Part C Coordinators (coordinator) serving as the designated state early intervention system leader for each participating state. (b) Agency administrator (administrator) participants had to have at least 1 year of experience leading an agency serving children with ASD under age 3 in a low-income region of the state, at an agency funded through the Part C system, and have at least one qualifying direct service provider also working at that agency. (c) Direct Service Providers (provider) met the following inclusion criteria: (1) having served at least 2 toddlers with high likelihood of ASD in the past year in a participating agency, and (2) having at least 1 year of experience with the population. (d) Primary Caregivers (caregiver) had the following inclusion criteria: (1) legal guardians of a child with or at high likelihood of having ASD participating in Part C services and (2) receiving services from a participating provider.

To facilitate a high response rate and obtain a broad view of publicly funded early intervention services for young children with ASD and their families in the US, participants were recruited from two primary sources. First, participants were recruited from the larger project's partners in 7 US states (i.e., Pennsylvania, New Mexico, Montana, Maine, Colorado, California, and Alabama). Participants originating from referrals through state partners consisted of approximately 27.4% of all survey participants. All other participants were recruited through a nomination system starting with state coordinators and ending with caregivers. State coordinators nominated at least two administrators in agencies providing early intervention services to low-resource and/or low-income families within their state. Participating administrators nominated at least three providers in their agency that directly serve young children with autism or high likelihood of autism. Finally, participating providers nominated at least one family on their caseload with a young child in this population. This method provided 72.5% of our total participant pool. If we did not get a response from at least one provider or one family, the study coordinator contacted the referral source to request an additional nomination.

The study team distributed online surveys via REDCap between November 2015 and April 2016 through email. To accommodate any technical or language barriers, arrangements were made to collect surveys from Spanish-speaking families over the phone. One survey was collected via postal service. Each participant received a survey-package specific to their role (coordinator, administrator, provider, caregiver). Participants were contacted by both phone and email with reminders to complete the survey and to answer any questions. Upon completion of the survey, participants were offered a $20 gift card for their participation.

### Respondents

One hundred and eighty one participants across 7 states were contacted, and 133 participated (73%). Participants included 8 state coordinators (88% response rate; two states had two coordinators complete the survey and one state did not complete the survey); 29 administrators (76% response rate), 57 providers (73% response rate), and 39 caregivers of children with autism (81% response rate). Seventy-three percent of all participants completed the survey, 29% remained unopened or unfinished, and 3% formally declined. See Table 1 for respondents' demographics.

**Table 1:** Participants and agency demographics.

**Variable**	**Coordinators**	**Administrators**	**Providers**	**Caregivers**	**Total**
	***M* (SD)**	***M* (SD)**	***M* (SD)**	***M* (SD)**	
Number of participants	*n = 8* (6%)	*n = 29* (22%)	*n = 57* (43%)	*n = 39* (29%)	*n = 133* (100%)
Child age (in month)	–	–	–	40.2 (21.2)	–
Participant's age (in years)	55.9 (4.5)	51.8 (10)	44.6 (12.5)	–	–
% Of children with ASD in agency	6.3 (4.7)	9.7 (9.3)	29.5 (31)	–	–
Years of experience with ASD	–	18.2 (11)	13.9 (9.3)	–	–
**Gender**					
Female	100%	90%	95%	100%	95%
**Ethnicity**					
Non-hispanic^*^	88%	86%	86%	79%	85%
Hispanic	12%	14%	12%	21%	15%
**Race**					
White	75%	90%	86%	77%	85%
Hawaiian/Pacific Islander	12%	3%	5%	8%	6%
Black/African American	12%	7%	5%	13%	8%
Asian	0%	0%	2%	0%	<1%
Amer Indian/Alaskan	0%	0%	2%	3%	1%
**Highest education**					
Some high school/HS/GED	0%	0%	0%	24%	7%
Some college	0%	0%	2%	29%	9%
College degree	25%	28%	32%	24%	28%
Master's degree	62%	62%	60%	16%	48%
Doctorate	12%	7%	0%	0%	2%
Other	0%	7%	3%	3%	5%
**Primary discipline**					
Psychologist	–	14%	14%	–	–
Marriage/family therapist	–	4%	2%	–	–
Social worker	25%	11%	7%	–	–
Speech therapist	–	4%	22%	–	–
Physical therapist	–	4%	–	–	–
Educator	63%	50%	31%	–	–
Behavior specialist	–	–	5%	–	–
Others	10%	14%	14%	–	–
**Marital status**					
Married	–	–	–	59%	–
Divorced	–	–	–	6%	–
Cohabiting, no marriage	–	–	–	13%	–
Single and unmarried	–	–	–	22%	–
Family annual income					
Under $25,000	–	–	–	26%	–
$25,000–$49,000	–	–	–	21%	–
$50,000–$74,999	–	–	–	15%	–
$75,000–$99,999	–	–	–	26%	–
$100,000 and above	–	–	–	13%	–

*The percentages not reaching 100% are due to minor-missing data*.

* *The terms Hispanic, Non-hispanic were used in the survey at the time. We use the more appropriate term Latinx in the manuscript*.

### Surveys

Surveys were chosen to characterize the early intervention service system context including service setting, funding, service intensity, parent/caregiver involvement and child needs, use and perspectives of EBP, and readiness for EBP implementation. Surveys asked about the types of intervention practices being used, including providers' perceived confidence using the interventions, and use of caregiver training method (e.g., psychoeducation/training, caregiver practice with feedback/coaching, etc.). Surveys included demographic questions, components of the ACT SMART Agency Assessment Battery (described below), and questions about implementation of new practices. Table 2 lists the surveys completed at each participant level.

**Table 2:** Participant survey completion.

**Participant Type**	**Survey components**
State Part C coordinators	Participant demographics Agency demographics ASD—Needs, Strategies and Context Survey (ASD-SIS)
Agency administrators	Participant demographics Agency demographics ASD—Needs, Strategies and Context Survey (ASD-SIS) Modified Practice Attitudes Scale (MPAS)—adapted
Direct providers	Participant demographics Agency demographics ASD—Needs, Strategies and Context Survey (ASD-SIS) Organizational Readiness to Change Assessment (ORCA) Texas Christian University Organizational Readiness for Change 4-Domain Assessment (TCU ORC-D4) Modified Practice Attitudes Scale (MPAS)—adapted
Caregivers	Participant demographics ASD-needs, strategies and context survey (ASD-SIS) Caregiver/client survey

#### Participant Demographics Survey

Participants at each level responded to questions describing their agency, experience and/or family. All participants provided information about their age, gender, race/ethnicity, and education. Caregivers responded to questions about marital status, income, and primary language spoken (one family completed the survey in Spanish) in the home. State coordinators, administrators and providers responded to questions about their primary discipline and years at the agency. Administrators and providers also indicated their years of experience working with youth with ASD and responded to questions about their training.

#### Agency Demographics Survey

Coordinators, administrators, and providers responded to questions regarding the percentage of children served in their agency/state had ASD, the service setting(s) and funding sources.

#### ACT SMART Agency Assessment Battery

This assessment battery, developed by Drahota et al. (36) specifically to provide a comprehensive, multi-level assessment of agencies providing services to children with ASD, compiles adapted versions of the ASD-Needs, Strategies and Context Survey (37), the Modified Practice Attitudes Scale [MPAS; (38)], the Organizational Context subscale of the Organizational Readiness for Change Assessment [ORCA; (39)], and TCU Organizational Readiness for Change-D4 [ORC-D4; (40)]. Measures were selected to evaluate the type and quality of intervention strategies and services being delivered within participating agencies and the extent to which services were perceived to be meeting client needs as well as organizational factors hypothesized to impact the quality and delivery of ASD services (e.g., communication within agencies; readiness for change; staff attributes, and attitudes) (41). A caregiver component was included to obtain perspectives on child and family needs, service provision and acceptability. Subscales from the following assessments were used in this study.

##### ASD-Needs, Strategies, and Context Survey

Participants across levels reported on areas of intervention need for children with ASD and how well a variety of needs were being addressed by the current system/agency, service intensity, and caregiver education and training methods. Administrators, providers, and caregivers were asked about the typical presenting problems of children with ASD or high likelihood of ASD. The specific need areas assessed included: *communication, social interaction, play, learning, sleep, eating, sensory, behavior challenges, stereotyped behaviors, repetitive and/or restrictive behaviors, parent-child engagement, and family stress around the child*. Administrators and providers also reported on the perceived effectiveness in addressing these needs on a Likert scale from: *not being addressed, not effective, somewhat effective, or very effective* *(**37**)*.

Additionally, participants across levels (including caregivers) indicated service intensity (number of hours per week), service location (home, school, community, childcare, clinic) and caregiver involvement (e.g., participation in goal development, observation of providers, practice using strategies, feedback on use, etc.). To better understand parent/caregiver involvement, we defined *caregiver training* as observation of providers working with the child, reading materials/resources and/or discussing the intervention with caregivers and *caregiver coaching* as providing the parents opportunities to practice a specific strategy with feedback and specified at-home practices between visits.

The measure also assessed which, if any, autism-specific practices were being used within the agency as perceived by administrators and providers. The listed items included evidence and non-evidence-based practices including 26 therapeutic strategies or interventions specific to ASD services for early intervention. Strategies were adapted slightly to include only those strategies appropriate for early intervention settings (e.g., intervention packages such as cognitive behavioral therapy and social skills training and treatment strategies such as cognitive restructuring were removed). Strategies and packages were listed by name in alphabetical order with no definition or information about their evidence base. Direct providers were further asked to rate their level of confidence in delivering any treatment strategies they said they reported utilizing on a Likert Scale (“I feel confident in my delivery of this practice”: *1–Disagree Strongly; 5–Agree Strongly)*.

##### Organizational Readiness to Change Assessment (ORCA)–Organizational Context

The ORCA-Organizational Context Scale (39) assessed organizational culture, defined as “normative beliefs and shared behavioral expectations in an organizational unit” (43 p. 770). Specifically, the ORCA measures staff perceptions of the quality of the organizational context to support practice change and innovation. The scale is comprised of six subscales: *leadership culture* (i.e., norms and expectations regarding how leaders behave and how things are done at the agency), *staff culture* (i.e., norms and expectations regarding how staff behave and how things are done at the agency), *leadership practices* (i.e., staff perception of leadership behaviors), *measurement* (i.e., staff perception of supervisor feedback), *readiness to change among opinion leaders* (i.e., performance measures and procedures for feedback and accountability), and *resources to support practice change*. Subscales consists of three to six items and all items are scored on a 5-point Likert scale from 1 (strongly disagree) to 5 (strongly agree). Scale and subscale scores are calculated by dividing the total score by the number of items on the scale resulting in scale score values of 1–5. Average scores below 3.8 are considered areas in need growth, while scores between 4 and 5 are considered areas of strength (i.e., 3.8–3.9 indicate an average score). Reliability tests indicate that the ORCA context subscale tool meets standard requirements of 0.80. Cronbach's alpha for reliability at 0.85.

##### Texas Christian University Organizational Readiness for Change 4-Domain Assessment

The ORC-D4 (40, 42) measures organizational climate, defined as the “way people perceive their work environment” across four major domains comprised of 21 scales and 125 items and [(43). p. 769]. Specifically, this measure assessed staff perceptions of their role in the organization. This project used the Staff Attributes (Growth, Efficacy, Influence, Adaptability, Satisfaction) Scale. For Staff Attributes, *growth* measures the extent to which staff value and perceive opportunities for professional growth; *efficacy* measures staff confidence in their own intervention skills; *influence* is the willingness and ability of staff to influence coworkers (be an opinion leader); *adaptability* is the ability for staff to adapt to a changing environment, and *satisfaction* examine overall job satisfaction. ORC-D4 scores have been associated with *higher satisfaction with training, greater openness to innovations* (44, 45), and better client functioning (42, 46). Response categories for the items on the ORC-D4 are on a 5-point Likert scale from 1 (strongly disagree) to 5 (strongly agree). Scale scores are computed by averaging scale items and multiplying by 10 to obtain a range of 10–50. A score of 30 indicates the scale's mid-point (neither agreeing nor disagreeing). Thus, scale scores above 30 indicate greater agreement and scale scores below 30 indicate greater disagreement with the construct. For Staff Attributes, scores above 40 are in the 75th %tile and considered a strength, excepting the *efficacy* scale which requires a score of 44.

##### Modified Practice Attitudes Scale

Adapted from the longer Evidence-Based Practices Attitude Scale (EBPAS) measure, the MPAS assessed attitudes toward treatment manuals specifically (47). The 8-item MPAS assessed both direct provider (consistent with original measure) and administrators (e.g., items were modified to reflect administrator attitudes toward providers use of EBP) attitudes toward EBP. A sample item includes: “[I am willing to OR I am willing to have clinical staff] use new and different types of interventions if they have evidence of it being effective.” Participants indicated their agreement with each item from 0 (*not at all*) to 5 (*to a very great extent*). The total score ranges from 0 to 40, and higher scores reflect more favorable attitudes toward use of EBP with scores above 32 indicating this as an area of strength. Scores below 22.5 indicate an area for growth in an organization. The cronbach's alpha for the MPAS was 0.80 in the original measure development study (27). For the current survey responses, the MPAS alpha coefficient maintained an 0.80 (38).

### Data Analysis

Data analyses were conducted using the SPSS 23.0 statistical software program. The characterization of early intervention services in the US was examined using descriptive statistics and mean difference analyses (i.e., Chi-square tests and independent samples *t*-test). Concretely, Chi-square tests (through contingency tables based on Bonferroni *post-hoc* method) were conducted to identify the discrepancies across the participant groups (i.e., administrators, providers, and caregivers) on intervention intensity, type of parent/caregiver training, coaching given and received, and the presenting needs of children with ASD. Two-tailed independent sample *t*-tests were used to detect discrepancies between administrators and providers on the perceived effectiveness of their team at addressing child's needs. To examine intervention practices and strategies used, and organizational variables associated with readiness to implement evidence-based practices, we conducted descriptive statistics, *Pearson* correlational analyses, and multiple linear regression analysis (i.e., using a backward elimination method to determine best model fit).

## Results

### Early Intervention Services in the US: Characterizing Services for ASD

#### Service Setting

The most frequent early intervention setting was the home (over 85% of all participants). The second most common setting was the community (coordinator = 37.5%, administrator = 67.9%, provider = 58.6%). Very few children received services in a clinic (<15%), and ~25% received services in school or daycare settings. We did not collect information about specific type of school or daycare setting or opportunities to interact with typically developing peers.

#### Methods Used for Therapeutic Goals

The most frequent method to establish intervention goals was through collaboration with caregivers. Respondents across levels similarly reported that early intervention goals were based on child and family needs (72.3%). Other methods included observing the child's behaviors and skills (53.6%) and using a specific assessment (44.1%).

#### Private vs. Public Funding Source

Based on agency report, 96.4% of the interventions provided to children with ASD were publicly funded (Part C), with a smaller percentage of interventions paid for privately (i.e., insurance, private pay, employer supported).

#### Intensity

All groups of participants agreed that about half of the children received fewer than 6 h of intervention per month. Administrators reported that only 18% of children received more than fifteen hours a month, while direct providers reported 25%, and caregivers reported that 43% received more intensive services (see Table 3). Because most services were provided in home (85%) it is likely these were provided using a one-to-one provider/child ratio. Surveys did not ask about caseloads. Although caregivers descriptively reported higher-intensity services, a Chi-square test showed that the differences reported by the three groups (i.e., administrators, providers, and caregivers) were not significant [$\chi_{\left(4\right)}^{2}$ = 6.27, *p* = 0.180].

**Table 3:** Intervention intensity reported by each participant.

**Intensity per month**	**Administrator %**	**Provider %**	**Caregiver %**
	**(*n* = 28)**	**(*n* = 48)**	**(*n* = 35)**
Fewer than 6 h	50	50	43
From 6 to 15 h	32	25	14
More than 15 h	18	25	43

When stratifying caregivers' report by family income, children from lower income families (< $50,000/year) were most likely to receive fewer than 6 h of intervention per month (*n* = 10, 56%). Children from families with higher income (> $50,000/year), were more likely to receive more than fifteen hours per month of intervention (*n* = 10, 59%). For more detailed information see Table 4. A Chi-square test analysis stratifying intensity of intervention by those families getting fifteen or less hours per month and those getting more than fifteen hours per month [$\chi_{\left(1\right)}^{2}$ = 3.44, *p* = 0.064] revealed marginally significant differences.

**Table 4:** Intervention intensity by income (caregivers).

**Intensity per month**	**Lower income %**	**Higher income %**
	**(*n* = 18)**	**(*n* = 17)**
Fewer than 6 h	56	29
From 6 to 15 h	17	12
More than 15 h	28	59

*Under $50,000 is referred to as low income, while $50,000 and above is referred to as high income based on median income in the US in 2016 being $59,039 (48)*.

#### Caregiver Training and Coaching

**Figure 1:**
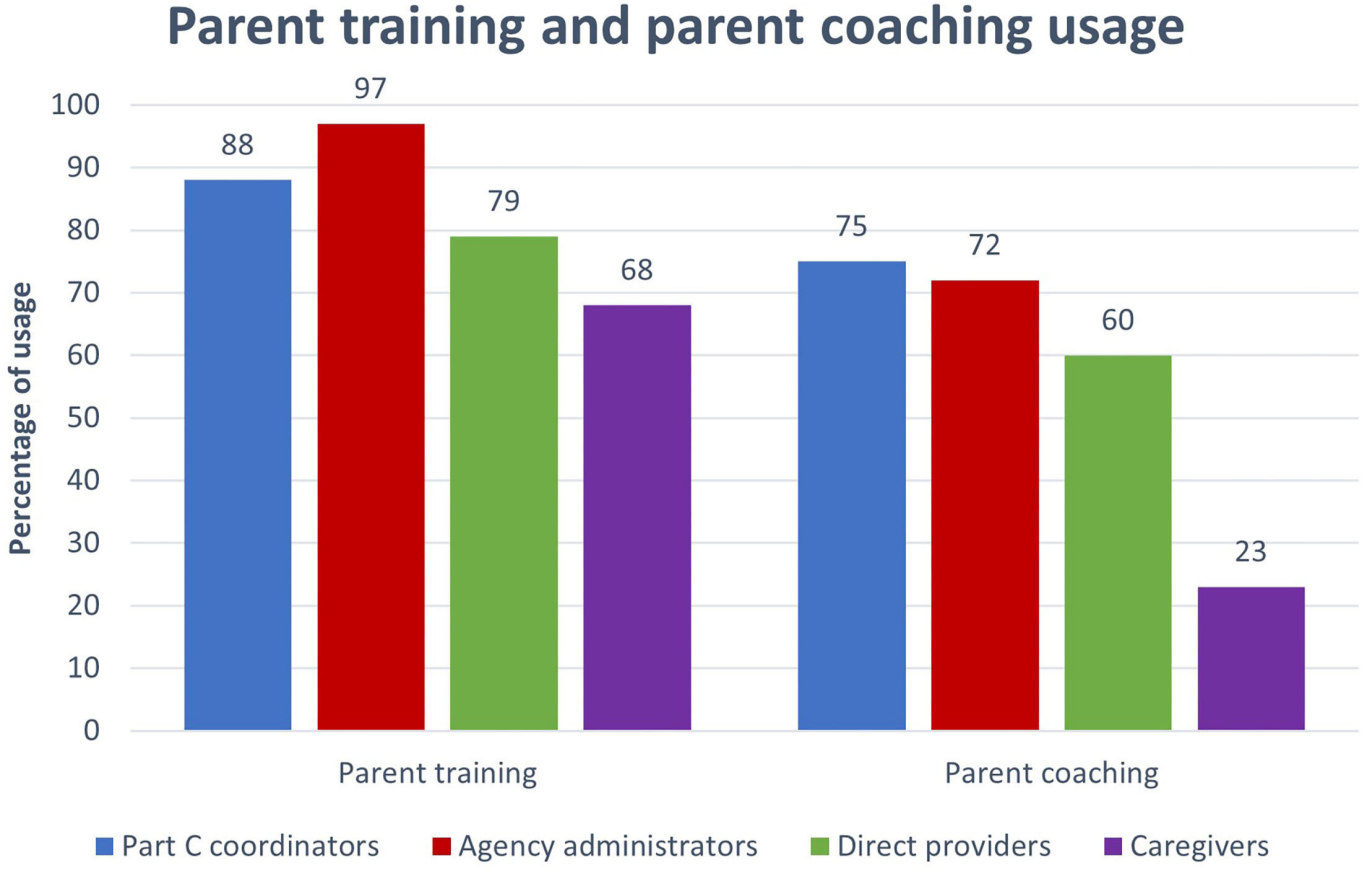
Parent coaching and parent training usages reported by all participants. Parents coaching refers to at-home practice together with feedback on parents use of strategies.

We asked participants about the use of caregiver training and caregiver coaching in early intervention. All participants reported high rates of caregiver training (68–97%; see Figure 1) while reported rates of coaching varied (23–75%). Chi-square test showed significant differences [$\chi_{\left(3\right)}^{2}$ = 20.95, *p* < 0.001] between groups of participants with regards to the reported frequency of caregiver training and caregiver coaching usage. While coordinators, administrators, and providers reported a high use of caregiver coaching strategies (over 60%), caregivers reported very low rates (23%). Providers indicate high rates of caregiver education and lower rates of caregiver coaching. However, caregivers did not report receiving training and coaching as often as the program staff reported (see Figure 1).

#### Presenting Needs of Children With ASD in Early Intervention

Administrators, providers, and caregivers were asked about the typical presenting problems for children with or at high likelihood of having ASD. All participant groups agreed the most frequently identified needs were addressing the development of communication, social interaction, play skills, concerns related to sensory differences, and behavioral challenges. All these areas were reported as key areas of need by over 80% of participants, regardless of participant role.

Chi-square tests were conducted to examine differences between the three groups (i.e., administrators, providers, and caregivers) in the reported areas of need. Results showed an overall agreement among the three participant groups in the reported areas of needs for young children with ASD. However, significant differences were found [$\chi_{\left(2\right)}^{2}$ = 11.97, *p* = 0.003] between the caregivers and both administrators and providers. Caregivers reported significantly higher levels of concern about *learning differences* (91%) compared to both professional groups (i.e., 50% administrators and 70% direct providers). See Table 5 for more details.

**Table 5:** Presenting client needs: children with ASD in early intervention.

	**Respondents**		
**Needs**	**Administrators %**	**Providers %**	**Caregiver %**
	**(*n* = 28)**	**(*n* = 54)**	**(*n* = 32)**
Communication skills	100	90	100
Social interaction skills	100	90	100
Play skills	93	92	100
*Learning differences*	50_a_	70_a_	91_a, b_
Parent-child engagement	75	52	47
Sleep challenges	68	60	66
Eating differences	71	75	94
Sensory differences	89	83	88
Behavior challenges	86	80	84
Stereotyped behavior	75	65	69
Family stress	75	72	84

*Variable in italics had significant differences across groups*.

*The different subscripts (a,b) refer to significant differences across those specific groups*.

#### Perceived Effectiveness of the Interventions Addressing Client Needs

Overall, descriptive analyses showed that administrators and direct providers reported feeling *somewhat effective* or *very effective* in addressing the needs of children with ASD. Communication skills were the only area where both administrators and direct providers reported the highest effectiveness (i.e., over 50% of both groups reported feeling *very effective*). There were four areas in which both groups felt less effective. More concretely, <25% reported feeling *very effective*, addressing *sleeping, eating, stereotyped/repetitive behaviors, or family stress*.

#### Comparing Agency Administrators' and Direct Providers' Perceptions of Early Intervention Effectiveness

A Student *T* test was conducted to identify differences between administrator and provider perceptions of effectiveness in addressing the developmental needs of children with ASD. Results indicated significant differences between administrators and direct providers in two areas: *social interaction* [*t*_(78)_ = 2.21, *p* = 0.03] and *stereotyped behaviors* [*t*_(67)_ = −2.27, *p* = 0.04]. More specifically, providers reported better skills in addressing social skills than was perceived by administrators. The opposite views were reported regarding stereotyped behaviors. That is, administrators perceived higher effectiveness of early intervention for addressing stereotyped behavior than providers.

#### Association Between Client Needs and Effectiveness Addressing Those Needs

When we combine these two sources of information, the presenting needs, and the effectiveness of the interventions addressing those specific needs, results showed that most administrators and direct providers reported feeling *somewhat effective* or *very effective* addressing client's highest needs (i.e., communication skills, social interaction, play skills, sensory differences, and behavior challenges). Administrators and direct providers disagreed about the effectiveness of intervention for *social interaction*. While over 50% of direct providers reported feeling *very effective* in supporting development of social interaction, only 28% of the administrators felt that way.

### Intervention Practices and Strategies for ASD utilized in Early Intervention

Administrators and providers reported on the practices used in their programs. Table 6 shows the proportion of participants reporting the use of a particular practice or strategy. Determination of the level of evidence for each strategy was based on the *Evidence Based Practices for Children, Youth, and Young Adults with Autism Spectrum Disorders Report* (2014) and the group's review of comprehensive treatment models (49). The 2014 review was used, rather than the 2020 update, to examine provider use of EBP identified at the time of the survey.

**Table 6:** Percentage of leaders and providers who report using practices/strategies in early intervention and provider's reported competence.

**Practice/strategy**	**Administrator strategy use %**	**Provider strategy use %**	**Provider reported high competence %**
	**(*n* = 28)**	**(*n* = 65)**	**(*n* = 65)**
**Evidence-based practice**
Reinforcement/rewards	75	75.4	95.9
Modeling	89.3	73.8	89.6
Visual supports (schedules)	82.1	69.2	86.6
Prompting	67.9	63.1	92.7
Alternative communication systems (e.g., PECS, sign, devices)	89.3	63.1	70.7
Parent-implemented intervention	60.7	53.8	73.5
Responsive teaching DIR/Floortime	35.7	46.2	96.5
Functional behavior assessment	39.3	46.2	65.5
Pivotal response training—naturalistic	53.6	44.6	82.8
Differential reinforcement	17.9	41.5	74.1
Positive behavior support (PBS)	35.7	41.5	92.6
Task analysis	14.3	41.5	76
Discrete trial teaching	28.6	40.0	73.1
Antecedent-based Intervention	25.0	40.0	84.6
Extinction	14.3	35.4	65.2
Social-communication intervention (e.g., SCERTS, Project ImPACT)—parent implemented	21.4	33.8	63.6
Early start denver model	35.7	32.3	33.3
**Emerging evidence**	
Sensory diet^*^	46.4	49.2	65.7
Expressive language-based therapy (e.g., HANEN)	32.1	47.7	87.1
Sensory integration^*^	75.0	47.7	48.4
Imitation-based intervention/reciprocal imitation training	28.6	41.5	74.1
Joint-attention intervention/instruction (e.g., JASPER)—naturalistic^*^	25.0	35.4	56.5
Music therapy	17.9	21.5	57.1
**No evidence to support**	
Play therapy	35.7	49.2	83.9
Dietary changes	28.6	33.8	54.5
Massage/touch therapy	35.7	24.6	56.3

* *Considered emerging evidence at the time of the survey by the NAEYC report*.

*Competence's columns indicate percentage of providers indicating feeling competent on this particular practice/strategy*.

Most administrators and providers endorsed many different practices. A similar proportion of providers reported using evidence-based practices, practices with emerging evidence, and those with no/limited evidence (i.e., EBP = 58.1%, emerging evidence = 55.4% and no evidence = 48.1% based on the 2014 report). All providers endorsed using at least 3 (out of seventeen) EBP. Providers tended to endorse strategies that addressed specific behaviors rather than comprehensive interventions addressing multiple areas of development.

#### Provider Competencies

Over 75% of providers *agreed* or *strongly agreed* that they felt competent delivering the following evidence-based practices and strategies: reinforcement/rewards (95.9%), modeling (89.6%), visual supports like schedules (86.6%), prompting (92.7%), responsive teaching DIR/Floortime (96.5%), pivotal response training—naturalistic (82.8%), positive behavior support (92.6%), task analyses (76%) and antecedent-based intervention (84.6%), see more details in Table 6.

#### Provider Training

The majority (76.3%) of providers indicated they received training through their school and/or educational coursework. However, over 50% of providers reported a need for more training in the following areas of competency: (a) ASD-related training (70.1%); (b) improving behavioral management of clients (59.7%); (c) improving engagement of caregivers during the session (56.1%); (d) increasing participation in interventions by clients with ASD or their families (54.4%); and (e) caregiver coaching strategies or methods (50.9%).

### Provider Readiness to Implement EBP in Early Intervention Programs

#### Attitudes Toward EBPs

MPAS scores did not differ by respondent type. Administrators, overall, had an MPAS mean score of 31.53 (*SD* = 4.90) and providers had a mean score of 30.79 (*SD* = 5.08) indicating this is an area that could benefit from additional growth and training across administrator and provider levels.

#### Organizational Context

Providers indicated that their early intervention agencies had average leadership culture (as indicated by ORCA scores; Table 7) for innovation implementation. They considered leadership practices, staff culture, and opinion leaders (at the staff level) as strengths in their organizations in terms of readiness to support the use of new practices. Measurement (i.e., leadership feedback on the use of intervention practices) and having resources to support practice change were both areas of need in early intervention agencies.

**Table 7:** Organization readiness to change (ORCA)—context scale.

**Subscale (*n* = 52 providers)**	**Mean (SD)**	**Rating**
Leadership culture	3.96 (0.79)	Average
Staff culture	4.27 (0.59)	Strength
Leadership practices	4.00 (0.82)	Strength
Measurement (leadership feedback)	3.75 (0.79)	Growth/Need
Readiness to change (opinion leaders)	4.42 (0.63)	Strength
Resources to support practice change	3.64 (0.86)	Growth/Need

#### Provider Attributes

Providers in early intervention agencies considered the attributes of staff in their agencies as strengths on two ORC-D4 staff attribute subscales (see Table 8): influence (i.e., staff interaction based on sharing and mutual support) and satisfaction (i.e., general satisfaction with one's job and work environment). They rated staff as average in the areas of growth, efficacy, and adaptability which may indicate that staff do not highly value or make use of opportunities to advance their own professional growth, may have poor confidence in their ability to deliver interventions or conduct their work well, and feel they have limited ability to effectively integrate new innovations at their agency.

**Table 8:** TCU Organization Readiness for Change (ORC-D4)—staff attributes scale (*n* = 52 providers).

**Subscale**	***M* (*SD*)**	**Rating**
Growth	39.90 (0.50)	Average
Efficacy	41.79 (0.44)	Average
Influence	40.12 (0.59)	Strength
Adaptability	39.13 (0.48)	Average
Satisfaction	44.82 (0.57)	Strength

#### Organizational Factors Associated With Attitudes Toward EBPs by Providers

Results showed a moderate positive association between provider attitudes toward EBPs and EBP usage (*r* = 0.39, *p* = 0.01). However, there was no association between attitudes toward EBPs and the provider's perceived competence using the EBP (*r* = 0.07, *p* = 0.61).

Regarding organizational factors, correlation analyses showed that provider attitudes toward EBPs were significantly associated with three of the organizational scales, leadership culture (*r* = 0.38, *p* = 0.01), staff culture (*r* = 0.28, *p* = 0.04), and growth (*r* = 0.39, *p* = 0.01), with low-to-moderate positive associations. That is, higher leadership culture, staff culture, and growth were related to more favorable attitudes toward EBP practices and strategies.

A linear regression analysis (i.e., backward elimination method) was conducted to assess whether organizational factors were related to the attitudes toward EBPs. Results of the linear regression model were significant, showing that ~31% of the variance in attitudes toward EBP was explainable by *readiness to change, leadership culture, resources to support practice change*, and *growth*, [*F*_(4, 47)_ = 5.40, *p* = 0.001, *R*^2^ = 0.31]. *Leadership culture* was significantly associated with attitudes toward EBPs, *B* = 2.70, *t*_(47)_ = 3.17, *p* = 0.003. This indicates that on average, a one-unit increase of *leadership culture* (as measured by the ORCA) was associated with increased attitude toward EBPs (MPAS) by 2.70 units. *Growth* (measured by the ORC-D4) was significantly associated with attitudes toward EBPs, *B* = 0.43, *t*_(47)_ = 3.29, *p* = 0.002. This indicates that on average, a one-unit increase of *growth* was associated with an increased attitude toward EBPs (MPAS) of 0.43 units. Table 9 summarizes the results of the regression model with the best model fit.

**Table 9:** Results for linear regression for attitudes toward EBPs.

**Variable**	***B***	***SE***	**95% CI**	**β**	***t***	***p***
(Intercept)	14.95	6.13	[2.61, 27.29]	0.00	2.44	0.019
Readiness to change	−1.86	1.09	[−4.05, 0.33]	−0.23	−1.71	0.094
Leadership culture	2.70	0.85	[0.98, 4.41]	0.42	3.17	0.003
Resources to support practice change	−1.09	0.76	[−2.61, 0.44]	−0.18	−1.44	0.158
Staff attributes growth	0.43	0.13	[0.17, 0.70]	0.43	3.29	0.002

*Results: F_(4, 47)_ = 5.40, p = 0.001, R^2^ = 0.31*.

## Discussion

This study examined three aspects of current early intervention practices for ASD to identify routes to improve translation and implementation of EBP in US publicly funded community early intervention settings. First, we sought to characterize the services delivered across seven states serving families in low-resource areas of the US. We found high levels of agreement across stakeholders in terms of the service setting, intensity, and needs of children entering the community early intervention system. Agency stakeholders and caregivers reported contrasting information about the extent of caregiver coaching delivery, with few caregivers reporting receipt of high-quality in-person coaching with their child. Interestingly, caregiver report of psychoeducation closely aligned with providers' rates of reported caregiver coaching. Over 50% of participating providers reported the need for more training in caregiver coaching strategies. Second, we specifically examined the current use of EBP in the system. The vast majority of providers reported using multiple strategies, about half of which could be considered evidence-based. While many providers felt competent in their delivery of several EBPs, endorsed strategies were specific rather than comprehensive, and 70% indicated the need for additional training in specific EBPs for ASD. Third, we examined organizational and contextual factors influencing system readiness for EBP implementation. Our data support a positive link between attitudes toward EBPs and EBP usage. Leadership culture and staff attribute growth were positively associated with providers' attitudes toward EBPs, pointing to contextual factors as potential leverage points to intervene upon to increase EBP use in community early intervention settings.

The US regulations for Individuals with Disabilities Education Improvement Act of 2004 (13) include several guidelines for Part C that support a “natural environment” for intervention, often interpreted as being the child's home for very young children, the use of scientifically-based interventions, and building early intervention competence within the child's family, including family involvement in goal setting and intervention delivery. No specific recommendations are provided for service intensity.

Our data indicate that a vast majority of children receive Part C services in the home or another community setting and very few are going to a clinic for services. This is very consistent with Part C regulations. Although no clear data exist to determine the specific intensity needed for early ASD services (11), general consensus in the field recommends at least ten hours of comprehensive treatment per week (10). Our data indicate that most children with ASD residing in low-resourced areas of the US receive ~6 h of intervention per month (i.e., fewer than 2 h per week) through publicly funded early intervention services. Providers responses indicated these services were predominantly a mix of individual strategies, rather than comprehensive, integrated programs. Moreover, low-income families reported receiving fewer service hours per month overall than higher-income families and the number of hours reported by low-income families aligned with Part C providers' overall report of service intensity. This suggests that higher-income families may be supplementing public early intervention services with additional intervention hours funded through insurance or self-pay methods. Higher-income families may also be able to use advocacy to garner more hours from the public system. To improve equity in provision of care, identifying equity-focused implementation strategies and allocation of services will be key to prevent widening disparities in access to needed services. However, ensuring the use of high-quality intervention in community programs may be even more critical given that poorly implemented interventions are not likely to improve child outcomes regardless of intensity.

Understanding the use of EBP in early intervention may provide some information regarding quality. Although to date, no specific intervention model or method has been established as the general standard for early intervention for ASD, many EBPs leading to gains in social communication, language, adaptative behavior, and learning have been identified (3–6, 49, 50). Some EBPs focus on specific skills and behaviors while others are applied across a range of skills and behaviors (50, 51). Both targeted and comprehensive strategies may need to be adapted to work within various public early intervention delivery systems (e.g., increased feasibility).

According to our results, most administrators and providers endorsed delivering multiple practices to youth with ASD, some with evidence and some with no/limited evidence. These results are consistent with prior studies in which providers report using an eclectic approach, combining different practices and strategies according to their personal criteria, typically in an unsystematic manner (25, 34). Providers reported competence in delivering several focused EBPs such as using rewards, modeling, prompting, and using visual supports. While they reported some confidence using a few complex EBPs (e.g., pivotal response training), fewer providers reported skills to deliver comprehensive interventions. This is consistent with observational studies indicating more accurate use of more structured, less complex interventions (52). However, comprehensive interventions may result in stronger outcomes (10, 52). Additionally, consistent with reports of limited coaching by caregivers, fewer providers reported confidence in their ability to effectively use caregiver coaching strategies or caregiver-implemented interventions, indicating a need for additional training or EBP adaptation to fit the system of care.

This lack of confidence in caregiver coaching may be a primary reason most caregivers in our sample reported receiving psycho-education rather than active, direct coaching with feedback, even though providers reported providing coaching. This discrepancy in reported use of caregiver coaching and training between administrators, providers, and caregivers is a common finding in publicly funded service provisions. For example, Straiton et al. (53) found a similar discrepancy between early intervention providers and caregivers in Michigan; providers who reported utilizing caregiver training were not typically endorsing EBP that aligned with caregiver training but rather psychoeducation and parental check-in strategies. Several factors could explain the discrepancy between caregivers and providers, including pre-service exposure to child and family-guided interventions (30). Some providers may use these terms and approaches synonymously. There may also be discipline-specific differences among coaching techniques where some may be more educational/structural than family-centered (54). Finally, caregiver expectations of intervention structure could also play a role in how they perceived coaching within the early intervention system.

Interestingly, another area of agreement among stakeholders included providers and administrators reporting challenges related to addressing eating, sleeping, family stress, and stereotyped behaviors presented by children with ASD in the early intervention setting. Other than stereotypy, these challenges involve associated but not ASD-specific behaviors, linked to higher levels of parenting stress, suggesting the need for targeted trainings in these areas. However, consistent with other studies, administrators and providers differed in reports of client needs, practice use and effectiveness of practices (34, 55). These discrepancies may imply a mismatch between provider training needs and training opportunities provided by administrators. Further, since providers are unlikely to communicate directly with state autism coordinators, it may be that individuals who can facilitate policy or funding for training are not aware of the support needed for providers to be able to meet the needs of their clients.

Organizational readiness to adopt and utilize new practices is critical for successful implementation of EBPs within an organization (56). Organizational readiness consists of motivation to try new practices, general capacity within an organization to support new practices, and necessary innovation-specific capacities, such as knowledge, skills, and resources. Both individual perspectives on the organizational climate as well as perspectives about the organizational norms are necessary to evaluate the capacity of an organization to deliver a new EBP at both the leader and provider levels (42). Organizational culture is a complex and dynamic set of constructs that coalesce to form an overall culture of readiness to implement an EBP (33).

Consistent with our finding that leadership characteristics relate to provider attitudes toward EBP, the implementation literature has established *leadership* as a key component for the successful adoption, implementation, and sustainment of EBP (57). Rather than considering only how we can provide adequate professional development to providers, we must also consider how to train leaders who support and recognize providers in their pursuit of improving high quality services (58).

In general, our data suggest that early intervention agencies could benefit from improved leadership climate and culture for innovation. While providers indicated some organizational strengths, such as the influence of opinion leaders, satisfaction with their work environment and culture for innovation at the provider level, they also reported challenges with consistent measurement of practice use, obtaining resources and feelings of efficacy and adaptability. Additionally, both administrators or providers reported relatively average or neutral attitudes toward EBP in general. The fact that attitudes toward EBP were associated with greater use of EBP highlights the importance of a focus on agency and provider buy-in to improve research-based early intervention services. Organizational context, in turn, explained much of the variance in EBP attitudes, indicating that a multi-level intervention addressing implementation readiness at the system, agency, and provider levels may be key to improving the quality of public early intervention programs (28, 58).

### Limitations

Our study has several limitations that should be noted when interpreting the results. First, although we sought diverse, representative stakeholders by focusing recruitment in states serving low-resourced areas (e.g., rural) which resulted in our caregiver sample having a range of incomes represented and 21% of caregivers identified as Latinx, a majority of our sample identified as white and non-Latinx. Additionally, most of the caregivers were married, with little representation of unmarried and single parents (i.e., 22%). Further, the administrator and provider samples are reflective of the limited diversity of the population. For instance, the American Speech-Language-Hearing Association (ASHA), the major professional organization for speech-language pathologists, reported that in 2020 96.3% of SLP members were female and 81.0% were white (59). Second, although we used a nomination system to connect the different participating layers to allow us greater consistency in understanding agreements and disagreements between participants, this complex recruitment method resulted in a small total sample size, limiting our statistical power and resulting in a less diverse sample overall.

### Recommendations for Practice

Our findings describe the early intervention features in low-resourced areas of the US, which could help future research translating EBPs into community programs to improve access to effective intervention for toddlers with ASD and their families. Given the limited resources of the system and low intensity of services, ensuring high-quality and model-adherent EBPs is especially important for families who cannot afford to pay for hours over and above what the public system provides.

The early intervention system itself and the agencies that provide early intervention services funded through the US Part C system would benefit from leadership training that supports implementation and sustainment of EBP, including caregiver coaching. Scientists have developed leadership training specific to supporting high-quality use of EBP (28, 58); testing these training programs in early intervention settings has the potential to increase access to quality care. Understanding how to create an organizational context and culture that values, supports, and rewards EBP use could drive low-resourced community services toward a general and effective use of EBPs, even in low-intensity services. Changing organizational culture is likely to influence provider attitudes toward EBPs, which in turn may additionally promote use of these EBPs and increase access to scientifically-supported interventions for more children and families.

Attitudes and practice can also improve through professional training. Professional training facilitates the use of caregiver training and coaching (53). Our results clearly suggest a majority of providers would welcome training in caregiver coaching as well as other specific EBP for autism symptoms and related concerns (e.g., sleep, feeding).

The best treatment response for young children occurs when early intervention combines both clinician- and caregiver-implemented components (60). Therefore, training in EBP and caregiver coaching designed to fit the early intervention context could boost providers' general capacity to implement best practices, including increased use of collaborative interventions involving primary caregivers. Long-term outcomes are stronger when there is an active participation of caregivers in the early intervention program (8). In low-resourced environments where low-intensity treatment hours are more likely to be delivered, a child's day-to-day learning opportunities rely more on caregiver use of EBP than children receiving high-intensity treatment. Thus, caregiver active involvement in intervention delivery is crucial to optimize learning opportunities and facilitate positive child outcomes (61). To meet this goal, providers need specific training in how to engage and coach caregivers successfully, how to support caregivers in integrating EBP into daily routines and activities, and how to adapt strategies to meet the individual needs of the family and child with ASD (62).

## Data Availability Statement

The raw data supporting the conclusions of this article will be made available by the authors, without undue reservation.

## Ethics Statement

The studies involving human participants were reviewed and approved by Institutional Review Board (IRB) for the University of California, Davis, project ID: 780328-22. The patients/participants provided their electronic informed consent to participate in this study.

## Author Contributions

AA: contributed to study conceptualization, methodology, fieldwork, and leading statistical analyses and interpretations of results, writing the original text, and contributing my own postdoctoral funding. AS: contributed to conceptualization, methodology, project multisite administration and supervision, obtaining project funding, and completing original writing. MT: contributed to the field work management, project administration, supervision, and significant review and editing of the manuscript. MM: contributed to project administration and fieldwork as well as manuscript editing. AD: provided study measures, contributed to results interpretation, provided original text, and edited manuscript content. MP: facilitated recruitment and project administration at one site, provided original text, and contributed to reviewing and editing the manuscript. AB: contributed to recruitment and project administration at one site, reviewed and edited the manuscript. EG: contributed by overseeing the fieldwork, local recruitment and administration of the project at one, and grant/manuscript editing. EM: contributed to recruitment at one site, survey development, and manuscript editing. SR: contributed to the study conceptualization, methodology, obtaining project funding, administering and supervising the project, and editing the document. All authors contributed to the article and approved the submitted version.

## Funding

This project was primarily funded through a U.S. Department of Education Research and Development Award (R324A150211). We received infrastructure support through the MIND Institute Intellectual and Developmental Disabilities Research Center (P50HD103526). Additionally, AA received funding for his work on the project through a Postdoctoral Fellowship of the Mas Casadevall-La Caixa Foundation for Autism Research ID20140714S7. MT's time was funded by the National Center for Advancing Translational Sciences, National Institutes of Health, through grant number UL1 TR001860 and linked award KL2 TR001859.

## Conflict of Interest

The authors declare that the research was conducted in the absence of any commercial or financial relationships that could be construed as a potential conflict of interest.

## Publisher's Note

All claims expressed in this article are solely those of the authors and do not necessarily represent those of their affiliated organizations, or those of the publisher, the editors and the reviewers. Any product that may be evaluated in this article, or claim that may be made by its manufacturer, is not guaranteed or endorsed by the publisher.
